# Executive function and spatial abilities in physically active children: an explorative study

**DOI:** 10.1186/s40359-024-01785-8

**Published:** 2024-06-04

**Authors:** Noemi Passarello, Patrizia Turriziani, Fabio Lucidi, Laura Mandolesi

**Affiliations:** 1 General and Experimental Psychology Laboratory, Department of Humanities, University of Naples “Federico” II, Naples, Italy; 2 Neuropsychology Lab, Department of Psychology, Educational Sciences and Human Movement, University of Palermo, Palermo, Italy; 3 Department of Social and Developmental Psychology, Faculty of Medicine and Psychology, University of Rome “Sapienza”, Rome, Italy

**Keywords:** Physical activity, Physical exercise, Ecological task, Cognition, Active lifestyle

## Abstract

**Background:**

Regular physical activity has consistently shown promise in improving cognitive functioning among children. However, there is a shortage of comprehensive studies that delve into these benefits across various cognitive domains. This preliminary investigation aimed to discern potential disparities in cognitive performance between active and sedentary children, with a specific focus on inhibitory control, cognitive flexibility, and visuo-spatial working memory abilities.

**Methods:**

The study employed a cross-sectional design encompassing 26 children (mean age 9.53 ± 2.20 years), categorized into two groups: Active and Sedentary. Executive functions were assessed using the NEPSY-II, while visuo-spatial working memory abilities were evaluated through the table version of the Radial Arm Maze (table-RAM) task. All outputs were analyzed with One-way ANOVAS or Kruskal–Wallis Tests to assess differences between Active and Sedentary children in both executive functioning and visuo-spatial working memory processes.

**Results:**

The findings revealed that the Active group outperformed the sedentary group in inhibitory control (F1,23 = 4.99, *p* = 0.03*), cognitive flexibility (F1,23 = 5.77, *p* = 0.02*), spatial span (F1,23 = 4.40, *p* = 0.04*), and working memory errors (F1,23 = 8.59, *p* = 0.01**). Both spatial span and working memory errors are parameters closely associated with visuo-spatial working memory abilities.

**Conclusions:**

Although preliminary, these results offer evidence of a positive link between physical activity and cognitive functioning in children. This indicates the importance of promoting active behaviors, especially within educational environments.

## Introduction

The effects of physical activity on the brain, cognitive functioning, and overall well-being have been extensively researched and documented. The accumulated evidence indicates that physical exercise not only enhances behavior and cognitive functioning, while also mitigating the risk of neurological diseases, safeguarding the brain against age-related decline, aiding in post-injury recovery, and boosting self-efficacy and self-esteem [1]. These aspects have been mostly studied in adults, with a specific focus on individuals engaged in both competitive and amateur sports [2, 3]. Numerous studies consistently demonstrate that experienced athletes exhibit heightened cognitive abilities, particularly in attention and cognitive flexibility domains, compared to novices and sedentary individuals [4, 5]. In this context, several researchers have proposed the hypothesis that expert athletes may have greater neural efficiency in their brains. This theory suggests that certain cognitive processes, particularly those related to action planning, are performed with minimal neuronal activation, resulting in optimal performance outcomes [6]. Recent evidence indicates that even adults engaging in regular physical exercise, without reaching competitive performance levels, demonstrate superior skills in specific cognitive tasks. Notably, they excel in areas such as task-switching and motor imagery tasks compared to individuals who do not engage in physical activity [7, 8].

Studies on the elderly consistently demonstrate that maintaining an active lifestyle can reduce cognitive decline, contributing to what is defined as “successful aging” [9, 10]. Whereas, in pre-adolescents, physical activity primarily influences executive functions and attention processes [11]. The positive effects of physical activity on executive functions can be attributed to neuroplastic changes in the central nervous system resulting from repeated and regular sports experiences [7]. While findings consistently highlight the beneficial impact of physical activity on the younger population, there remains considerable ambiguity in understanding its effects on specific cognitive domains [12]. Notably, research conducted on children, pre-adolescents, and adolescents has predominantly focused on the relationship between sport practice and academic achievements [13, 14]. Active children demonstrate improved attention in the classroom, grasp verbal information quickly, and excel in abstract reasoning and mathematical tasks compared to their sedentary peers [12]. However, further research is needed to better understand how physical activity could impact specific cognitive domains, such as executive function and visuo-spatial abilities. Additionally, it’s important to note that cognitive assessments in children mostly use traditional pen-and-paper tests, despite a growing trend toward digitalization. These assessment methods may not always reflect real-world ecological scenarios, where the interaction between the individual and their environment plays a crucial role in cognitive processes. This aspect has been somewhat overlooked in current research [12, 15]. Our preliminary study aims to investigate potential differences in cognitive performance between active and sedentary children, with a specific focus on inhibitory control, cognitive flexibility and visuo-spatial working memory. We tested a sample of children (mean age 9.53 ± 2.20), categorize as Active and Sedentary, based on their activity level. To assess executive abilities, we utilized the NEPSY-II [16], while the table version of the Radial Arm Maze (table-RAM) [17] to evaluate physical exercise effects on visuo-spatial working memory.

Both the NEPSY-II and the table-RAM task represent an ecologically valid approach to assess cognitive functions in children. This approach improves the reliability and significance of our findings and enables a thorough analysis of various aspects within these cognitive domains.

## Material and methods

### Participants

The study comprised a sample of 26 healthy children, including 6 males, with a mean age of 9.53 ± 2.20 years. The participants were divided into “Active” and “Sedentary” based on their level of physical activity (Table 1).

**Table 1:** Participants’ demographics. Age ± SD are reported

	**No**	**Age (years)**	**Education (years)**	**Sport experience (years)**	**Sport frequency (times/week)**
Active	13	8.02 ± 0.76	3.07 ± .86	2.66 ± .12	1.2 ± .31
Sedentary	13	7.76 ± 0.24	2.74 ± .32	0	0

The participants in the Active group had a minimum of 3 consecutive years of experience in their sport (primarily martial arts) and engaged in regular practice at least twice a week. On the other hand, the participants in the Sedentary group had no history of regular and consistent participation in any sports. This information was obtained from the interview with the parents or legal guardians who, after being informed about the study, signed the written informed consent (see “Ethical statement” section).

Inclusion criteria for participation in the study were normal or corrected-to-normal vision and right-handedness. Exclusion criteria encompassed the presence of current or past neurodevelopmental disorders, neurological or motor disorders, or any significant medical illnesses.

### Ethical statement

This study received approval from the Local Ethics Committee of the “Federico II” University of Naples (protocol number: 14/2023) and followed the principles outlined in the Declaration of Helsinki. Written informed consent was obtained and signed by the parents of all participants.

### Measures

#### Executive functions assessment: NEPSY-II

The NEPSY-II is a commonly used assessment paper and pencil tool for evaluating executive functions in children and preadolescents [18, 19]. The NEPSY-II provides a thorough evaluation of multiple cognitive domains, such as attention, inhibition, working memory, cognitive flexibility, and planning. In this study, we utilized the inhibition task from the NEPSY-II to assess cognitive flexibility and self-regulation. Specifically, we measured the ability to suppress or inhibit automatic responses. The task consisted of three phases: Denomination, Inhibition, and Switching. During the Denomination phase, participants were required to correctly label the figures they saw based on specific instructions (e.g., “square” for a square figure, “circle” for a circular figure). In the Inhibition phase, participants had to inhibit their previously learned response and provide the opposite label for the figures (e.g., saying “circle” for a square figure and vice versa). Finally, in the Switching phase, participants were instructed to switch the rule they had previously acquired and provide a different label for the figures based on a new instruction (e.g., saying “square” for a black square and “circle” for a white square).

For each phase, the experimenter recorded on the schedule the time by means of stopwatch, and the number of errors.

#### Visuo-spatial working memory assessment: table version of the Radial Arm Maze task (table-RAM)

**Fig. 1:**
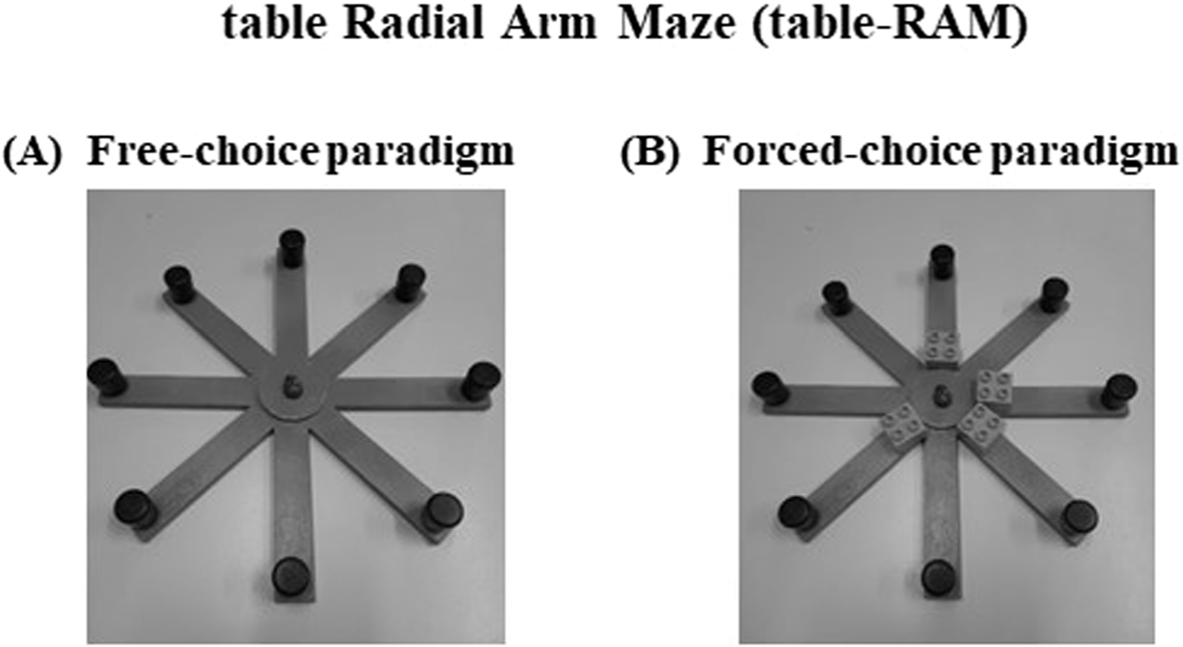
Table version of the Radial Arm Maze (table-RAM): **A** free-choice paradigm; **B** forced-choice paradigm (phase 1)

Table version of the Radial Arm Maze task (table-RAM) [12, 17] was utilized to assess visuo-spatial working memory abilities. Table-RAM’s apparatus comprised a round central platform with a diameter of 5 cm. Eight green arms, each measuring 3 cm in width and 25 cm in length, extended from the central platform resembling spokes of a wheel (Fig. 1). At the end of each arm, a small black round cap with dimensions of 1 cm in diameter and 2 cm in height covered a reward, represented by a colored wooden ladybug. The table-RAM was placed on a desk, while the extra-maze cues, such as windows, paintings, posters, doors, and the experimenter, were maintained in constant spatial relationships throughout the experiment. The participants were only allowed visual access to the table-RAM during the assessment.

The table-RAM task was presented to the children as the “Ladybug game”. The aim of the game was to move the main ladybug, positioned on the central platform, to find its hidden sisters located within the caps at the end of each arm. To enhance motivation, children received a reward (a coin) after completing each trial by discovering the ladybugs. The children were evaluated using two different RAM versions: the free-choice paradigm, which analyzed the peripersonal procedural and memory components, and the forced-choice paradigm, which aimed to distinguish the components associated with visuo-spatial working memory from the procedural ones. The participants’ choices during each trial of both paradigms were recorded through video recording and manual registration.

##### Free-choice paradigm

In this paradigm, the children were allowed to freely explore the eight arms to find the ladybugs hidden inside the caps at the ends of each arm (Fig. 1A). A trial concluded when all eight ladybugs were collected, 20 choices were made, or after 5 min from the start of the task. Since each cap contained one ladybug, optimal performance entailed visiting each upturned cap only once, resulting in eight visits to the arms. This can be best achieved by creating 45° angles between near-located arms (e.g., visit arm 2, then 3, then 4, and so on). Revisiting the same arm within the same trial was considered an error. The experimenter provided the same simple verbal instructions at the beginning of the first trial to explain the task to each participant. No further instructions or verbal encouragement were given once the task started. Participants’ performance in the free-choice paradigm was evaluated based on the following parameters: *Search Efficiency* (the percentage of wrongly visited arms out of the total entries made), *Spatial Span* (the longest sequence of correctly visited arms, ranging from 1 to 8), and *Strategy Efficiency* (the percentage of 45°degree angles out of the total number of angles made).

##### Forced-choice paradigm

The day after the free-choice paradigm session, children were tested in the forced-choice paradigm. In the first phase, although all arms contained ladybugs, only four arms (e.g., arms 1, 3, 4, 6) were accessible, while the remaining four arms were randomly closed using Lego cubes (2 cm × 2 cm × 2 cm) placed at the proximal ends of each arm (Fig. 1B). Different angles were used to separate the opened arms, preventing participants from reaching a solution using procedural strategies, such as only visiting adjacent angles. The task started with the main ladybug placed on the central area of the maze. Children were allowed to explore the four open arms by moving the main ladybug to collect the accessible ladybug sisters. Subsequently, children were asked to pause the game and were relocated to a separate area where they could not see the maze. They engaged in a conversation with the experimenter for 120 s before the second phase of the task began. The experimenter asked all the participants what fun they had done the day before and during this short conversation the labyrinth was not visible to them because their backs were turned. In the second phase, children were allowed to move the main ladybug in all arms, but only the four previously closed arms were rewarded (as the other four ladybugs had already been collected in the previous phase). Success in visiting only the rewarded arms primarily depended on remembering which arms had already been visited, emphasizing the memory component while neglecting search patterns. At the beginning of the trial, the experimenter provided the same simple verbal instructions to explain the task to each participant. After the 120-s interval, the verbal instructions were given as follows: “Uh! Something has changed; there are no Lego bricks anymore. Now, the ladybug can freely search for the other sisters!”.

In the forced-choice paradigm, the parameter considered was the number of working memory errors divided by the total number of entries (*Total WM Errors*), including across-phase errors (visits into an arm previously visited during the first phase of the same trial; *Across-Errors*) and within-phase errors (re-visits into an arm already visited in the same phase; *Whitin- Errors*). The longest sequence of correctly visited arms (*Spatial Span*), in this case ranging from 1 to 4, was also considered in the analysis.

### Study design

The study employed a cross-sectional design to investigate the relationship between physical activity and cognitive performance in children. Participants were recruited prior to an interview with their parents or legal guardians to gather information about their physical activity levels. Based on this information, participants were categorized into two groups: Active and Sedentary, following the criteria described in “Participants” section. After collecting descriptive information, the testing procedure was conducted over two separate days. On the first day, participants underwent assessment of executive functions using the NEPSY-II. Subsequently, on the following day, participants completed the table version of the Radial Arm Maze (table-RAM) task, which included both the free-choice and forced-choice paradigms. A 2- hours pause was incorporated between the two versions of the table-RAM task to minimize fatigue and maintain participant engagement. During both testing sessions, data were collected by a trained researcher who provided standardized instructions and monitored the children’s performance closely to ensure consistency in testing procedures across participants. 

For NEPSY-II, participants performed one trial for each phase of the task, with initial training [18, 19]. For the table-RAM participants performed one trial for each paradigm (free-choice and forced-choice). All data collections were carried out in a silent room with only the experimenter inside with the children. The all procedure took about 30 min for each task.

### Data analysis

To assess demographic differences between groups, independent sample t-tests were conducted to compare age and years of instruction, while chi-squared test was employed to examine gender differences. For NEPSY-II raw scores were compared with normative mean and SD to exclude any participant who showed clinical scores [20].

To test the hypothesis regarding differences in executive functions between the Active and Sedentary groups, one-way ANOVAs were performed. Group (Active vs. Sedentary) was entered as the factor variable, and each NEPSY-II parameter (Errors, Time) was used as the dependent variable. Separate analyses were conducted for each of the three trials: Denomination, Inhibition, and Switching.

Similarly, to investigate the hypothesis regarding differences in spatial abilities between the groups, one-way ANOVAs were conducted. Group (Active vs. Sedentary) was used as independent variable, while each free-choice and forced-choice parameter served as the dependent variable. 

Z-scores were calculated for both the NEPSY-II and RAM outputs to deal with outlier participants. The normality of the data was assessed using the Shapiro–Wilk’s test, and non-parametric analyses (such as Kruskal–Wallis’s test) were employed when the data deviated from a normal distribution. The equality of variance was evaluated using Levene’s test, and if violated, Brown-Forsythe’s correction was applied for homogeneity. Post-hoc comparisons were conducted using Games-Howell’s and Tukey’s tests, with a 95% confidence interval considered, including lower (LCI) and upper (UCI) bounds. Due to the relatively small sample size, post-hoc outcomes were bootstrapped from 1000 replicates.

All analyses and graph work were conducted on JASP version 0.17.1.0.

## Results

### Participants’ demographics

Independent sample t-test revealed no significant differences between Active and Sedentary for both age (t = 1.11; *p* = .27) and instruction years (t = 2.01; *p* = .70). Similarly, chi-squared test revealed no significant differences for gender (χ^2^ = .68;* p* = .41).

Additionally, it is worth noting that none of the participants exhibited NEPSY scores within the clinical range, as illustrated in Table 2.

**Table 2:** NEPSY-II Denomination, Inhibition and Switching trials score (time) for participants compared to normative data [20]

	**Normative** mean ± SD	**Active** mean ± SD	**Sedentary** mean ± SD
**NEPSY-II Denomination**	57.4 ± 9.8	67.25 ± 3.0	57.53 ± 10.15
**NEPSY-II Inhibition**	81.4 ± 17.8	86.58 ± 7.04	79.76 ± 8.82
**NEPSY-II Switching**	128.8 ± 29.6	127.58 ± 9.05	135.76 ± 9.63

### Differences between Active and Sedentary in executive domains

**Fig. 2:**
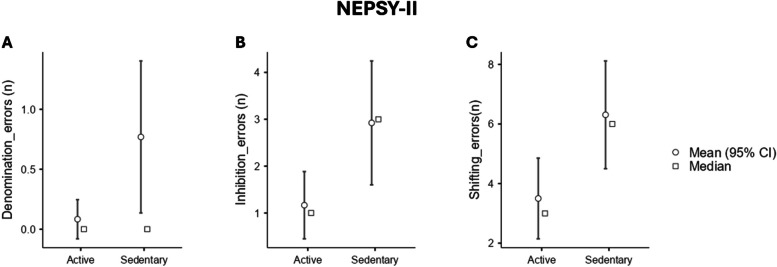
One-way ANOVAs output for executive function assessment through NEPSY-II. The number of errors is reported for each trial: **A** Denomination trial; **B** Inhibition trial; **C** Shifting trial. Abbreviations: * = *p* ≤ .05

The results of one-way ANOVAs indicated no significant differences between groups in the Denomination trial for both Time (K _1,23_ = 1.74, *p* = .20) and Errors (K _1,14_ = 4.22, *p* = .06) Table 3 and Fig. 2A). In the Inhibition trial, no significant difference was observed between groups for Time (K_1,23_ = .90, *p* = .35). However, a significant difference was found for Errors (K _1,23_ = 4.99, *p* = .03*). Bootstrapped (BS) post-hoc analysis using Games-Howell revealed that the Active group made significantly fewer errors compared to the Sedentary group (_BS_ Mean difference = -.82, _BS_LCI = -1.71, _BS_UCI = -.21, _BS_SE = .37, _BS_p = .03*) (Fig. 2B). Similarly, no differences were found for Time in the Shifting trial (K _1,23_ = .63, *p* = .43), but a significant difference was observed for Errors (K _1,23_ = 5.77, *p* = .02*). Games-Howell bootstrapped (BS) post-hoc analysis revealed that the Active group made significantly fewer errors compared to the Sedentary group (_BS_ Mean difference = -.87, _BS_LCI = -1.58, _BS_UCI = -.25, _BS_SE = .34, _BS_p = .02*) (Fig. 2C).

**Table 3:** Descriptive data for NEPSY-II Denomination, Inhibition and Switching trials (errors)

	**Mean**	**SD**
**Denomination**		
Active	.08	.29
Sedentary	.76	1.16
**Inhibition**		
Active	1.16	1.26
Sedentary	2.92	2.43
**Shifting**		
Active	3.50	2.39
Sedentary	6.31	3.32

### Differences between Active and Sedentary in visuo-spatial working memory abilities

**Fig. 3:**
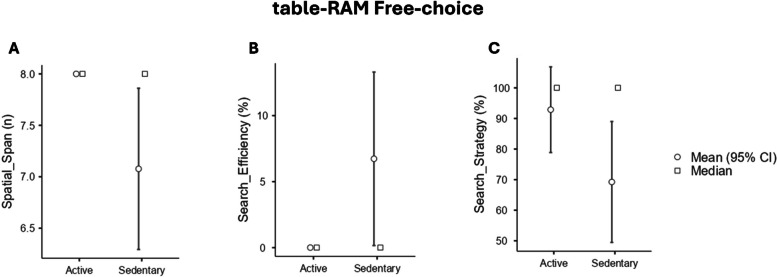
Kruskal–Wallis tests output for spatial abilities assessment through RAM free-choice paradigm. **A** Search Efficiency %; **B** Search Strategy%; **C** Spatial Span. Abbreviations: * = *p* ≤ .05

In the Free-choice trial, no significant differences were found for Search Efficiency (K _1,12_ = 4.03, *p* = .07) and Search Strategy (K_1,21_ = 3.66, *p* = .07) (Table 4, Fig. 3A and B). However, significant difference was found for Spatial Span (K _1,12_ = 5.33, *p* = .04*). Bootstrapped (BS) post-hoc Tukey’s test revealed that Active had a bigger span than Sedentary (_BS_ Mean difference = .84, _BS_LCI = .06, _BS_UCI = 1.63, _BS_SE = .38, _BS_p = .03*) (Fig. 3C).

**Table 4:** Descriptive data for table-RAM free-choice. Abbreviations: IQR = interquartile range

	**Median**	**IQR**
**Spatial Span**		
Active	8.00	.00
Sedentary	8.00	1.41
**Search Strategy**		
Active	100.00	.00
Sedentary	100.00	6.37
**Search Efficiency**		
Active	100.00	.00
Sedentary	100.00	5.74

**Fig. 4:**
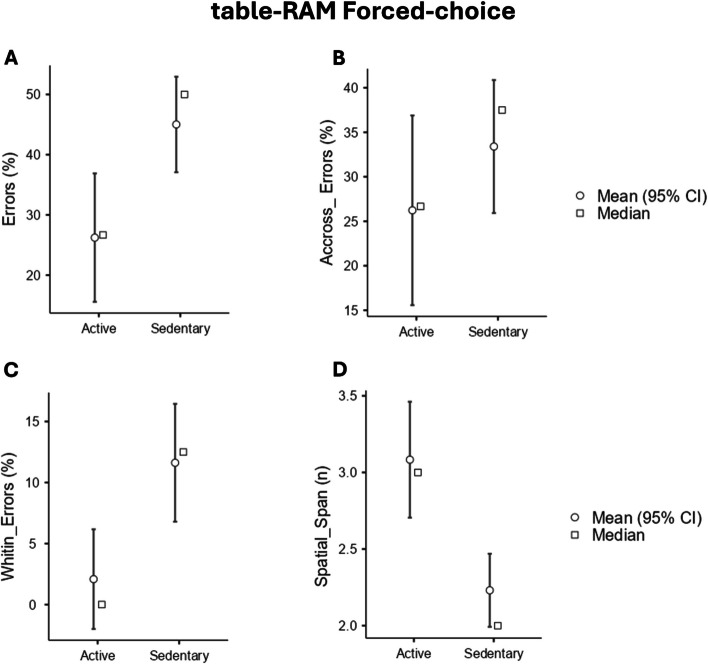
Kruskal–Wallis tests output for spatial abilities assessment through RAM forced-choice paradigm. **A** Total Working Memory Errors %; **B** Whitin-errors % **C** Across-errors %; **D** Spatial Span. Abbreviations: * = *p* ≤ .05; **p ≤ .01

As for the Forced-choice trial, significant differences were found for both Total Working Memory (WM) Errors (K _1,23_ = 7.85, *p* = .01**) and Within-Errors (K _1,23_ = 8.59, *p* = .01**). Bootstrapped (BS) Games-Howell post-hoc analyses revealed that Active did less general WM Errors (_BS_Mean difference = -.96, _BS_LCI = -1.84, _BS_UCI = -.41, _BS_SE = .35, _BS_p = .001**) (Fig. 4A, Table 5), an specifically less Whitin- Errors (_BS_ Mean difference = -1.05, _BS_LCI = -1.52, _BS_UCI = -.203, _BS_SE = .33, _BS_p = .01**) (Fig. 4C). No significant differences were found for Across-Errors (K _1,23_ = 1.20, *p* = .29) (Fig. 4B). Lastly, significant difference was found for Spatial Span (K_1,23_ = 4.40, *p* = .04*). Bootstrapped (BS) Games-Howell post-hoc analyses revealed that Active had bigger Spatial Span than Sedentary (_BS_ Mean difference = .79, _BS_LCI = .01, _BS_UCI = 1.46, _BS_SE = .38, _BS_p = .04*) (Fig. 4D).

**Table 5:** Descriptive data for table-RAM forced-choice. IQR = interquartile range

	**Median**	**IQR**
**Spatial Span**		
Active	3.00	.25
Sedentary	2.00	.00
**Total WM Errors**		
Active	26.66	20.86
Sedentary	50.00	7.14
**Across-WM Errors**		
Active	26.66	18.86
Sedentary	37.50	11.49
**WhitinWM Errors**		
Active	.00	.00
Sedentary	12.5	20.00

## Discussion

This preliminary research aimed to explore the beneficial impact of sports participation on executive functions and visuo-spatial working memory abilities in children. Our results showed that Active children performed better than those who were sedentary in tasks related to inhibitory control, cognitive flexibility, and visuo-spatial working memory.

Concerning executive functions, the Denomination trial of the NEPSY-II revealed no notable disparities in Time and Errors between the Active and Sedentary groups (Fig. 2A). This implies that the capability to accurately label figures didn’t show significant differences between the two groups. This outcome was anticipated since only healthy individuals were part of the sample (Table 2) [20].

Conversely, in the NEPSY-II Inhibition trial, which requires heightened engagement of executive functions like inhibition control, Active children showed similar execution times to Sedentary children but made fewer errors (Fig. 2B), indicating superior accuracy. These results align with previous research that highlights the positive impact of physical activity on inhibitory control in children [21–24]. Notably, there is compelling evidence indicating that even a single session of aerobic exercise can notably enhance inhibitory control performance in children with ADHD [25–28]. Likewise, research has shown the immediate effects of treadmill walking on inhibition control in preadolescents, with improvements observed in both inhibitory control and cognitive flexibility. [29, 30].

Lastly, in the NEPSY-II Shifting trial, which tests children’s cognitive flexibility, our results showed significantly fewer errors in the Active group compared to the Sedentary group (Fig. 2C). This finding closely supports previous research indicating a strong connection between acute physical activity and enhanced cognitive flexibility and set-shifting abilities in children [23].

The solid connection between physical exercise and cognitive flexibility, as evidenced in our study and supported by existing literature, can be explained by the ongoing improvement of this cognitive ability through sports practice. Whether in controlled indoor settings like gyms or dynamic outdoor spaces like playgrounds, the exercise environment constantly evolves [31–33]. This dynamic nature of exercise environments serves as training for individuals to adeptly adapt to changing contextual conditions [34, 35]. This observation underscores the idea that action itself is a form of cognition [36].

Significant findings also arise from participants’ performance in the table-RAM task In the Free-choice paradigm, there were no significant differences found between Active and Sedentary individuals regarding Search Efficiency and Search Strategy (Fig. 3A and B). However, notable differences emerged in Spatial Span, with the Active performing better than the Sedentary group (Fig. 3C). It’s worth noting that the free-choice paradigm is inherently straightforward and requires minimal engagement with procedural processes related to exploring peri-personal space. As a result, deviations in the Search Efficiency and Search Strategy parameters are primarily observed in pathological conditions [37].

On the other hand, the Forced-choice paradigm significantly elevates memory demands, making it a valuable tool for assessing working memory capabilities [38]. In our study, the Active group demonstrated fewer total Working Memory Errors (Fig. 4A) and Within-Errors (Fig. 4B). It’s essential to highlight that the Forced-choice paradigm enables the characterization of the “type” of working memory errors [17]. Across-Errors indicate visits to an arm previously accessed during the initial phase of the task, while Within-Errors signify revisits to an arm previously explored within the same phase. Therefore, Within-Errors are more difficult to make because it is easier to remember the arms in the same phase. However, in our study, sedentary individuals made a higher number of Within-Errors in comparison to Active children, suggesting thus how sports practice improves these memory processes. Moreover, Active children demonstrated significantly higher Spatial Span values (Fig. 4D) compared to the Sedentary group. These results are consistent with previous research emphasizing the positive impact of sports participation on visuospatial working memory across various age groups [12, 28, 30–41]. Supporting these behavioral findings, Erickson et al. [42] showed that aerobic exercise increased the volume of the hippocampus, a brain region crucial for spatial memory processing. Together, these findings strongly support the idea that engaging in sports activities can effectively enhance working memory capabilities.

In conclusion, this study adds value by employing assessment tools with greater ecological validity, enabling a more accurate and representative evaluation of children’s cognitive functions outside clinical or laboratory settings. One such tool is the RAM task, extensively utilized in various applications to assess cognitive functions in both typically developing populations and those with neurodevelopmental deficits [17, 38]. Its effectiveness lies in its unique ability to evaluate visuo-spatial working memory abilities by combining elements of a “game” with a comprehensive assessment of all aspects of spatial function [43].

## Limitations

Despite the valuable findings presented in this study, it is important to acknowledge certain limitations. One significant limitation is the small sample size, which may limit the generalizability of the results. A larger sample would provide a more representative picture of the population. Additionally, the study was unable to explore potential gender differences in visuospatial abilities, despite their known influence on spatial processing. Future research should aim to include a larger and more diverse sample to examine potential gender-specific effects.

Further, it’s worth noting that our present study concentrated on a particular age group and excluded participants with clinical diagnoses. Expanding the research protocol to encompass diverse populations in terms of age and clinical conditions would contribute to a more holistic comprehension of the influence of sports engagement on cognitive functions. Encompassing individuals with varying characteristics would facilitate the exploration of whether the identified effects hold true across distinct populations or if there exist specific subgroups that might derive greater advantages from sports involvement.

In our study, we did not utilize a quantitative, standardized tool to differentiate between active and sedentary children. Instead, we relied on classification criteria specified in the Methods section obtained from parents, this represents another limitation.

Another limitation concerns the fact that in this study, for reasons of availability, participants performed a single trial for free choice and forced choice. This does not allow us to evaluate learning processes and requires us to take the results obtained with caution.

Finally, it’s crucial to consider the potential for digitalizing tools, especially the Table-RAM task. Embracing digital platforms and technologies has the potential to enhance the real-world applicability of assessments, making them more interactive and accessible on a wider scale [44, 45]. Digitizing RAM and other cognitive tasks can simplify their administration, data collection, and analysis, ultimately improving the efficiency and scalability of research efforts [43].

## Conclusions

In conclusion, this preliminary study provides compelling evidence for the beneficial effects of sport participation on cognitive functions, specifically in the domains of executive functions and spatial abilities. Although our results must be considered with caution, given the various limitations, they align with previous research demonstrating the association between physical activity and improved cognitive performance [7]. An active lifestyle not only promotes physical health and well-being but also contributes to the holistic development of children by fostering social skills, discipline, and perseverance. Future studies should explore these aspects and further explore the potential of digitalization in cognitive assessments.

## Data Availability

No datasets were generated or analysed during the current study.
